# Measuring malaria transmission reduction en route to elimination

**DOI:** 10.1186/1475-2875-13-S1-O26

**Published:** 2014-09-22

**Authors:** Richard W Steketee

**Affiliations:** 1 Malaria Control and Elimination Program and MACEPA, PATH, Seattle WA, USA

## 

In the past decade, remarkable change in malaria control and transmission has been documented in many (but not all) African nations and some countries are moving toward elimination. The malaria control community has long measured and tracked malaria transmission using a variety of methods from the highest to the lowest transmission areas. For program action across the spectrum of transmission between humans and mosquitoes, the balance between accurate measurement, sufficient detail to inform program decisions, and ease and simplicity of information collection, analysis and interpretation has always been challenging. A summary of historic and current efforts to measure parasites within and moving between people and mosquitoes will be provided with an emphasis on how the approaches and emphasis change as transmission intensity changes. The program decision needs for routine systems and for special studies to track transmission reduction will be discussed. The detection and verification of “zero” infection and transmission will be discussed in terms of tools and sampling methods that will allow the designation of malaria-free areas.

